# Natural Exposure- and Vaccination-Induced Profiles of *Ex Vivo* Whole Blood Cytokine Responses to *Coxiella burnetii*

**DOI:** 10.3389/fimmu.2022.886698

**Published:** 2022-06-23

**Authors:** Susan Raju Paul, Anja Scholzen, Ghazel Mukhtar, Stephanie Wilkinson, Peter Hobson, Richard K. Dzeng, Jennifer Evans, Jennifer Robson, Rowland Cobbold, Stephen Graves, Mark C. Poznansky, Anja Garritsen, Ann E. Sluder

**Affiliations:** ^1^Vaccine and Immunotherapy Center, Massachusetts General Hospital, Boston, MA, United States; ^2^InnatOss Laboratories B.V., Oss, Netherlands; ^3^Sullivan Nicolaides Pathology, Brisbane, QLD, Australia; ^4^School of Veterinary Science, University of Queensland, Gatton, QLD, Australia; ^5^Australian Rickettsial Reference Laboratory, Geelong, VIC, Australia

**Keywords:** Coxiella burnetii, Q fever, vaccination, infection, cytokines signature, human, IFNgamma, IL-2

## Abstract

Q fever is a zoonotic disease caused by the highly infectious Gram-negative coccobacillus, *Coxiella burnetii* (*C. burnetii*). The Q fever vaccine Q-VAX^®^ is characterised by high reactogenicity, requiring individuals to be pre-screened for prior exposure before vaccination. To date it remains unclear whether vaccine side effects in pre-exposed individuals are associated with pre-existing adaptive immune responses to *C. burnetii* or are also a function of innate responses to Q-VAX^®^. In the current study, we measured innate and adaptive cytokine responses to *C. burnetii* and compared these among individuals with different pre-exposure status. Three groups were included: n=98 Dutch blood bank donors with unknown exposure status, n=95 Dutch village inhabitants with known natural exposure status to *C. burnetii* during the Dutch Q fever outbreak of 2007-2010, and n=96 Australian students receiving Q-VAX^®^ vaccination in 2021. Whole blood cytokine responses following *ex vivo* stimulation with heat-killed *C. burnetii* were assessed for IFNγ, IL-2, IL-6, IL-10, TNFα, IL-1β, IP-10, MIP-1α and IL-8. Serological data were collected for all three cohorts, as well as data on skin test and self-reported vaccine side effects and clinical symptoms during past infection. IFNγ, IP-10 and IL-2 responses were strongly elevated in individuals with prior *C. burnetii* antigen exposure, whether through infection or vaccination, while IL-1β, IL-6 and TNFα responses were slightly increased in naturally exposed individuals only. High dimensional analysis of the cytokine data identified four clusters of individuals with distinct cytokine response signatures. The cluster with the highest levels of adaptive cytokines and antibodies comprised solely individuals with prior exposure to *C. burnetii*, while another cluster was characterized by high innate cytokine production and an absence of *C. burnetii*-induced IP-10 production paired with high baseline IP-10 levels. Prior exposure status was partially associated with these signatures, but could not be clearly assigned to a single cytokine response signature. Overall, Q-VAX^®^ vaccination and natural *C. burnetii* infection were associated with comparable cytokine response signatures, largely driven by adaptive cytokine responses. Neither individual innate and adaptive cytokine responses nor response signatures were associated retrospectively with clinical symptoms during infection or prospectively with side effects post-vaccination.

## Introduction

Q fever is a zoonotic disease caused by an intracellular Gram-negative coccobacillus, *Coxiella burnetii* (*C. burnetii*) (1). It is predominantly transmitted to humans through inhalation of infected aerosols secreted by ruminants such as sheep, goat and cattle. *C. burnetii* is highly contagious with as few as 1-10 inhaled bacteria being sufficient for transmission of infection (2). Combined with the high stability of *C. burnetii* in the environment, this has resulted in the organism being classified as a Category B pathogen with potential for use as a biological weapon by the US Centers for Disease Control and Prevention (1, 3). To date the largest reported natural outbreak of Q fever was recorded in the Netherlands between 2007-2010, with an estimated 40,000 infections based on seroconversion at the centre of the epidemic area alone (4, 5).

The majority of individuals remain asymptomatic during acute infection with *C. burnetii* (6). When symptomatic the most common manifestation of acute Q fever is febrile illness, pneumonia or hepatitis (7). Clinical diagnosis is typically confirmed through whole blood PCR testing and/or through serologic testing (8). Acute Q fever is rarely fatal and is effectively treated by tetracyclines (7). However, persistent infection, also known as chronic Q fever, is diagnosed in 1-5% of acute cases and is commonly characterized by endocarditis, aneurysms or vascular infections (6, 8, 9). While these cases are often asymptomatic during initial acute infection, if left untreated chronic Q fever has a fatality rate of 60%.

Lipopolysaccharide (LPS) is the most well-known virulence factor for *C. burnetii* (10). *C. burnetii* LPS undergoes structural variation by truncation upon serial passage *in vitro*, also known as phase transition (11). LPS of freshly isolated virulent *C. burnetii* (phase I) contains LPS O-antigen, while serial passage leads to mutations in LPS biosynthesis genes, resulting in avirulent phase II *C. burnetii* lacking the terminal LPS O-antigen sugars (10, 12, 13). Clinically, phase II antibodies are detectable prior to phase I antibodies during acute infection, and the persistent presence of phase I antibodies differentiates acute from chronic Q fever (8, 14).

The only commercially available vaccine for humans is Q-VAX^®^, which is based on the whole cell formalin-inactivated phase I *C. burnetii* Henzerling strain (15). The vaccine is highly protective, but is licensed for use only in Australia (6, 7). This is largely due to the fact that Q-VAX^®^ vaccination can induce severe side effects in those with prior immunity to *C. burnetii*, and hence is only administered to individuals with both a negative humoral Q fever response and a negative hypersensitivity skin test (16, 17).

Humoral responses are initiated 7-14 days after acute infection with *C. burnetii* (7). However, about 20% of those infected become seronegative after 4-6 years, with an extrapolated half-life of 318 days for phase II IgG (18). Moreover, studies in animal models have shown that while antibodies are required to control tissue damage, they alone are not sufficient to control infection. Instead, in these animal models T-cells and in particular IFNγ and TNFα responses are critical for controlling early infection and mediating bacterial clearance (19–22). Cellular IFNγ responses and/or anti-*C. burnetii* IgG in peripheral blood can be detected in humans for up to 10 years post-vaccination with whole cell formalin-inactivated phase I *C. burnetii* Henzerling strain (23). A study of mostly elderly individuals who were offered Q-VAX^®^ after the Dutch Q fever outbreak found that pre-vaccination *C. burnetii*-specific IFNγ responses correlated with local reactions after skin test (17). However, whether these vaccine side effects in pre-exposed individuals are truly only associated with pre-existing adaptive immune responses to *C. burnetii* or are also a function of innate responses to Q-VAX^®^ components remains unclear.

In this study, we assessed which patterns of innate and adaptive cytokine responses to *C. burnetii* are induced upon *ex vivo* stimulation of whole blood. We compared how these cytokine patterns differ among groups with different pre-exposure status, namely unexposed individuals and those exposed by vaccination with Q-VAX^®^ or by prior infection with *C. burnetii*, and analysed whether cytokine responses had any relation to clinical symptoms experienced following vaccination or natural infection. Overall, Q-VAX^®^ vaccination and natural *C. burnetii* infection were associated with comparable cytokine responses that were largely driven by adaptive cytokines. Neither individual innate and adaptive cytokine responses nor response signatures were associated retrospectively with clinical symptoms during infection or prospectively with side effects post-vaccination.

## Materials and Methods

### Ethics Statement

The human study involving Dutch inhabitants of the village of Herpen, NL was reviewed and approved by the Medical Ethical Committee Brabant (Tilburg, Netherlands, NL74801.028.20) and all participants provided written informed consent.

The human study involving Australian veterinary students undergoing routine Q-VAX^®^ vaccination was reviewed and approved by the University of Queensland Human Research Ethics Committee (Brisbane, Queensland, Australia, protocol 2020001442) and all participants provided written informed consent.

### Human Study Cohorts

Samples from n=98 de-identified adult blood bank donors from the Amsterdam/Rotterdam area (The Netherlands) with unknown prior exposure to *C. burnetii* were obtained in November 2020 from the Sanquin Blood bank (**Table 1**) (24).

**Table 1:** Demographics of human study subjects.

Group	N	Age in years (median, min-max)	Females N (%)
Dutch village cohort	95	60 [26-80]	57 (60%)
Dutch blood bank cohort	98	45 [21-73]	48 (49%)
Australian pre-vaccination cohort	96	19 [18-39]	83 (86%)
Australian post-vaccination cohort	58	19 [18-32]	51 (88%)

Adults with known prior *C. burnetii* exposure status from earlier studies were recruited from the Dutch village of Herpen and surrounding areas, the epicenter of the 2007-2010 Q fever outbreak (25). All study participants in this cohort had previously been tested between 2014 and 2016 using the Q-detect™ IGRA to determine cellular immunological responses to *C. burnetii* (24). The majority of the participants enrolled in the present study were also assessed by immunofluorescence assay (IFA) for serological responses in 2014 (26) and followed up in more detail for cellular responses between 2015 and 2017 (27). In the latter study, preference was given to participants with strong responses to whole heat-killed *C. burnetii* in the IGRA to maximize the potential to detect *C. burnetii* epitope-specific T-cells. Therefore, the level of cellular responses in this cohort is not representative of all individuals with prior *C. burnetii* exposure. In total, n=95 Dutch adults with known past exposure status provided written informed consent and were available for blood collection in January 2021, 10-14 years after natural infection. This included n=84 individuals from the initial Q Herpen II study (26, 27) with known (positive or negative) IGRA and serology status, as well as n=11 individuals with known past symptomatic Q fever and positive IGRA status, but unknown prior serology status (**Table 1**). Of these 95 individuals, n=13 had no history of Q fever disease (26) and scored negative by IFA in 2014 (24) and 2021 (28) as well as by IGRA in 2014, 2015 and 2021 (24), and were designated as unexposed/no prior infection. Amongst the remaining n=82 participants with known positive IGRA in 2014 and 2015, n=81 had available information on clinical symptoms during past infection (either registered (notified) in the national surveillance system, or self-reported) (26, 27) and were subdivided into asymptomatic (n=47) or symptomatic (n=34).

Blood samples were collected from Australian veterinary students before and after undergoing routine Q-VAX^®^ vaccination between February and May 2021 (**Table 1**). Briefly, at enrollment student participants were screened for pre-exposure *via* serological analysis by enzyme-linked immunosorbent assay (ELISA) for phase II IgG, as well as a skin test using a 1/1000^th^ dose of the Q-VAX^®^ vaccine by intradermal injection. Three students had low-level positive phase II IgG ELISA results and were therefore additionally assessed but tested negative by a phase II IgG immunofluorescence assay (IFA; titer <1:25) and a complement fixation (CF) assay (titer < 1:2.5). An experienced physician visually assessed skin tests after 7 days. All n=96 students were eligible for Q-VAX^®^ vaccination based on a negative composite phase II IgG (ELISA, IFA and CF results), and the absence of skin test reactions. The majority of post-vaccination samples were collected within a target window of 28-35 days after vaccination (median 30 days) from n=58 students who attended the follow-up visit. However, short-term lockdowns to contain local clusters of SARS-CoV-2 infections resulted in some visits being outside the target window, with an overall range of 16-68 days for post-vaccination sample collection. Of those vaccinated, n=38 students did not return for the post-vaccination blood draw. Local and systemic reactions within seven days after skin test and vaccination were self-reported by students *via* an online survey administered through the SurveyMonkey platform (Momentive, Inc.).

### Whole Blood Stimulation With Heat-Killed *Coxiella* Antigen

Whole lithium-heparin anti-coagulated blood was stimulated on the day of blood collection with *C. burnetii* antigen (heat killed strain Cb02629), following the Q-detect™ protocol (24). The Cb02629 strain, isolated from the placenta of a goat infected during the Dutch outbreak, carries the phase I LPS variant. Cb02629 antigen was prepared from a master cell bank by a cell-free culture method using acidified citrate cysteine medium at the Central Veterinary Institute, Wageningen Bioveterinary Research, Lelystad, The Netherlands (29, 30). Recovered cells were washed repeatedly to remove cell media and then heat-killed. For the stimulation, microtubes were pre-coated with heat-killed whole cell *C. burnetii* Cb2629 antigen or a positive control, namely *Staphylococcus* enterotoxin B (Sigma, Cat. No. S4881). Both antigens were deposited in a sucrose matrix. The negative control stimulus for the assay was the sucrose matrix on its own. Stimulation was carried out by adding 250µL blood per stimulation tube. After 21-23 hours of incubation at 37°C, whole blood plasma supernatants were collected and IFNγ concentrations assessed using a fully validated in-house ready-to-use human IFNγ ELISA. All samples were assayed in a 4-fold dilution. Concentrations were calculated using a standard curve obtained by four parameter logistic curve fitting. Negative control responses that were too low to be calculated were assigned a concentration of 0.6 pg/mL, which is the limit of quantification of the ELISA. Concentrations above the range of the standard curve were assigned 105% of the concentration of the highest standard (500 pg/mL) multiplied by the dilution factor, resulting in an upper limit of quantification of 2100 pg/mL. The negative control cutoff was set at >40 pg/mL and positive control cutoff was set at <40 pg/mL. A subject was scored positive if negative and positive controls met the cut-offs, the *C. burnetii*-induced IFNγ production was ≥10 pg/mL above background and the stimulation index (SI; IFNγ conc.^(^*^C. burnetii^*^)^:IFNγ conc.^(negative control)^) of the *C. burnetii*-specific response was SI ≥10, while those with a SI ≥3 but <10 were judged as borderline as previously described (24). 11/347 individuals had borderline IGRA results and one individual amongst these was positive for phase II IgG by IFA.

In this specific study, IGRA results were not deemed inconclusive when the negative (n=7) (**Supplementary Figure 1A**) or positive (n=8) control (**Supplementary Figure 1B**) cut-offs of IFNγ production were not met. Instead, innate responses in the same *C. burnetii* and negative control stimulated samples, as determined by V-plex, were used as an indicator of sample viability. Samples were then judged as IGRA positive or negative solely based on *C. burnetii*-induced IFNγ level and SI criteria.

#### Multiplex Cytokine Secretion Analysis Following Whole Blood Stimulation

Plasma supernatants from whole blood stimulation cultures were collected and frozen for later multiplex cytokine analysis. Quantification of cytokines was conducted using a sandwich-ELISA based multi-spot electrochemiluminescence detection system from Meso-Scale Discovery, using the human Proinflammatory Panel 1 V-plex kit (IL-1β, IL-2, IL-6, IL-10 and TNFα) and the human Chemokine Panel 1 V-plex kit (CXCL-8/IL-8, CCL3/MIP-1α and CXCL-10/IP-10). Only supernatant samples from negative control and *C. burnetii* stimulated whole blood were assessed for these cytokines. Analysis was conducted following the manufacturer’s recommendations, with a 10-fold dilution of all samples and adjustment of the standard curve reconstitution volume to extend the standard curve range and hence the upper limit of quantification. Cytokine responses were background corrected per donor by subtraction (cytokine conc.^(^*^C. burnetii^*^)^ minus cytokine conc.^(negative control)^).

### Serological Responses to *C. burnetii*

For the Dutch cohorts, phase II IgG antibody titers were determined according to the manufacturers’ instructions by IFA (Focus Diagnostics) at the Jeroen Bosch Hospital, ‘s-Hertogenbosch, the Netherlands. Phase II IgG titers of ≥1:64 were interpreted as IFA-positive, consistent with the original Q Herpen II study (26, 28).

The pre-vaccination screening of the Australian veterinary student cohort was conducted using a NATA (31) accredited in-house indirect phase II IgG ELISA with microwells coated with the Nine Mile strain (ATCC 616-VR) and manufactured by Vircell S.L (Spain). In addition, pre- and post-vaccination serum samples underwent extended serological testing in parallel to assess phase II IgG and IgM responses by ELISA, phase I and phase II IgG, IgM and IgA by IFA using Vircell S.L (Spain) manufactured slides coated with *C. burnetii*, Nine Mile strain (ATCC 616-VR), and for CF antibodies to both phase I and II using the Virion/Serion (Germany) Complement Fixation Test system. IFA dilutions for IgG commenced at 1:100 while IgA and IgM commenced at 1:25. CF responses commenced at doubling dilutions from 1:2.5 as recommended for pre-vaccination screening. For the purpose of calling participants seropositive or negative by phase II IgG IFA, titers of ≥1:100 were interpreted as IFA-positive. However, in the n=3 cases when phase II IgG IFA was conducted to confirm or refute ELISA results during pre-vaccination screening, a cutoff of ≥1:25 was used.

### Computational Analysis and Data Visualization

Dimension reduction was performed on background corrected cytokine data after arcsinh transformation using Uniform Manifold Approximation and Projection (UMAP) (32) with the package umap-learn. Subsequently spectral clustering was performed on UMAP embeddings using the package sklearn. The optimal number of clusters was identified using the kneed algorithm as implemented in the package kneed. All analyses were done using Python version 3.8. Figures were created using GraphPad Prism v9 (San Diego, CA, US) and using packages Matplotlib (Version 3.5.1) and Seaborn on Python 3.8.

### Statistical Analysis

Statistical analysis was performed using GraphPad Prism v9 (San Diego, CA, US).

## Results

### Adaptive Cytokine and Antibody Responses Against Phase II *C. burnetii* but Not Innate Cytokine Responses Are Elevated Four Weeks After Vaccination

In order to study the induction of immune responses upon Q-VAX^®^ vaccination, we assessed whole blood cytokine responses to heat-killed *C. burnetii* as well as serological responses to phase I and phase II *C. burnetii* antigens both pre- and post-vaccination in a cohort of Australian veterinary students. Cellular responses were determined by IGRA and whole blood stimulation supernatants were additionally assessed for release of eight other innate and adaptive cytokines and chemokines.

**Figure 1:**
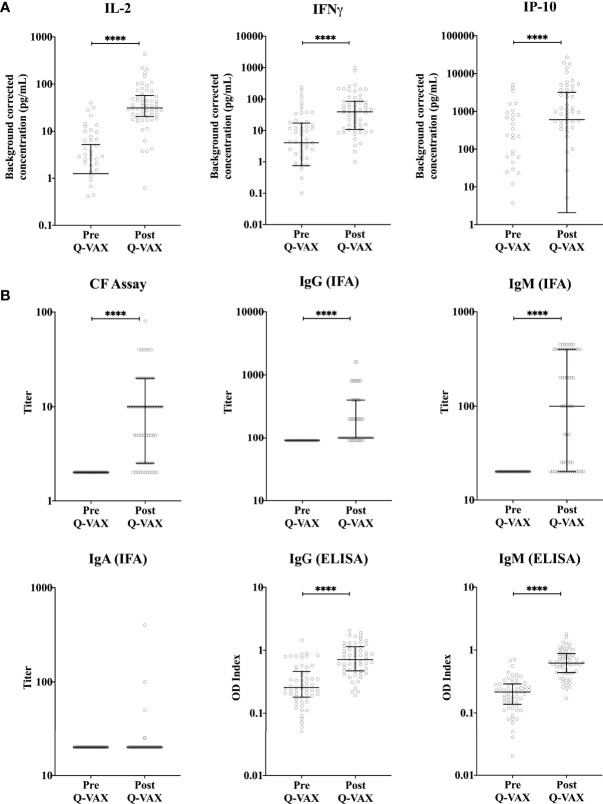
Vaccination-induced changes in cellular and humoral responses to *C. burnetii*. Cytokine release upon whole blood stimulation with *C. burnetii* and serological responses were determined for paired pre- and post-vaccination samples from n=58 subjects undergoing Q-VAX^®^ vaccination. Data are represented using a scatter dot plot. Lines and error bars show the median and interquartile range. Pre- and post-vaccination responses were compared using Wilcoxon matched-pairs signed rank test. The asterisks (****) indicate p ≤ 0.001. **(A)** Background corrected IP-10, IL-2 and IFNγ concentrations in whole blood supernatants prior to vaccination and at 4-5 weeks post-vaccination. Data are displayed on a log scale and hence zero and negative values (including medians and error bars in that range) are not represented in the graph. **(B)** Phase II IgG, IgM and IgA IFA titers, CF titers and IgG and IgM ELISA optical density (OD) index (OD^serum^: OD^cut-off^) in serum prior to vaccination and at 4-5 weeks post-vaccination.

Within the student cohort, 25% (24/96) were positive and 4% were borderline (4/96) by IGRA prior to vaccination, however none of the participants were positive for phase II IgG by IFA. Amongst the cytokines assessed, the adaptive cytokines IL-2, IFNγ and the IFNγ-induced protein-10 (IP-10) were significantly elevated four weeks post-vaccination (**Figure 1A**). Innate cytokines and other chemokine responses (IL-8, MIP-1α, IL-1β, IL-6, IL-10 and TNFα), in contrast, did not change significantly after vaccination (**Supplementary Figure 2**). Notably, the relative increase particularly for IFNγ (and IP-10) was greater and more consistent in participants who were IGRA negative prior to vaccination (**Supplementary Figure 3****).** 97% (34/37) of participants who were IGRA negative pre-vaccination had a 3-fold increase in IFNγ response after vaccination. However only 38% (8/21) of participants who were IGRA positive pre-vaccination had a three-fold increase in IFNγ response after vaccination. The six participants with the highest pre-vaccination *C. burnetii*-specific IFNγ response (60-240 pg/mL) showed either no clear increase or a decrease in IFNγ responses post-vaccination. Phase II IgG, IgM IFA titers and CF titers were significantly elevated post-vaccination, while phase II IgA IFA titers remained low at 4 weeks post-vaccination (**Figure 1B**). No IgG, IgA or CF responses were seen against phase I *C. burnetii*. although 2/58 participants had detectable phase I IgM IFA titers (1:50 and 1:200) at four weeks post-vaccination (data not shown). Correlation analysis between cytokines, antibodies, and days post-vaccination (Q-VAX^®^) revealed a positive correlation for IL-2 responses with IFNγ release, phase II IgG, IgM (IFA and ELISA) and CF antibody titers and the length of the interval between vaccination and sample collection for response assessment (days after Q-VAX^®^) (**Supplementary Figure 4**).

### Individuals With Prior Exposure Through Infection or Vaccination Show Comparable Adaptive but Not Innate Cytokine Responses

**Figure 2:**
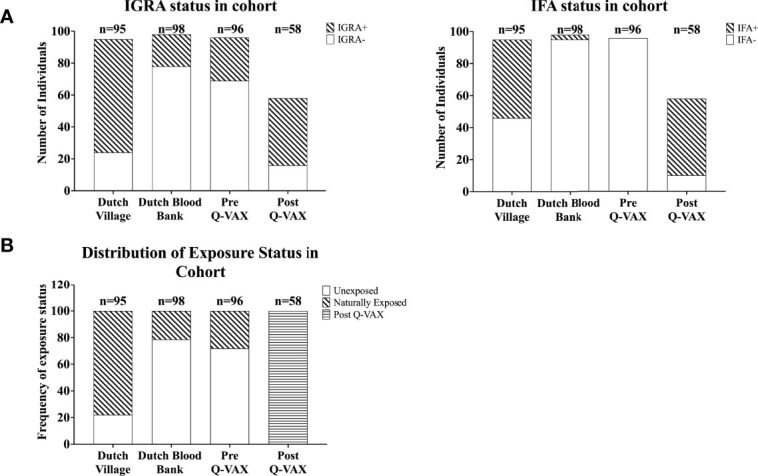
Categorization of individuals based on IGRA and IFA. Data is represented as stacked bar plots. **(A)** The absolute number of individuals positive and negative by IGRA (left) and for IgG against Phase II *C. burnetii* (right) in each cohort. **(B)** Frequency of exposure status (defined based on IGRA and IFA) within each cohort.

Next we compared how cytokine response patterns to *C. burnetii* differ between study participants that were exposed to inactivated *C. burnetii* by Q-VAX^®^ vaccination versus those that experienced a natural infection with viable *C. burnetii*. In addition to the vaccination cohort of Australian veterinary students, we also assessed *C. burnetii*-specific responses in 193 Dutch adults from the Sanquin blood bank cohort and the Dutch village cohort with different degrees of natural exposure to *C. burnetii*. In the Sanquin blood bank cohort where prior exposure to *C. burnetii* is unknown, n=20 (20% of cohort) were positive by IGRA (5/98 IGRA borderline and 15/98 IGRA positive) and n=3 had positive IgG antibody titers for phase II *C. burnetii* (3% of cohort). This was comparable to the Australian veterinary students with unknown exposure status prior to vaccination (29% IGRA positive and all sero-negative for Phase II IgG). The distribution and degree of C. *burnetii*-specific IFNγ responses were highly similar between the Dutch Sanquin blood bank cohort and the Australian students pre-vaccination cohort (**Supplementary Figure 1C**), consistent with minimal prior clinically-relevant *Coxiella* exposure in both groups, as reflected by the absence of positive skin tests in the students. Post-vaccination, n=43 participants tested positive by IGRA (74% of cohort) and n=48 had positive IgG antibody titers for phase II *C. burnetii* (83% of cohort). Only two of the vaccinees (3% of cohort) remained negative by both IGRA and phase II IgG IFA post-vaccination. In the Dutch village cohort, which includes a large proportion of individuals with known past exposure to *C. burnetii*, n=71 participants (75% of cohort) were IGRA positive and n=49 had measurable IgG antibody titers for phase II *C. burnetii* (52% of cohort). This distribution of cellular and humoral responses reflects the varying prior exposure status within each cohort (**Figure 2A**).

**Figure 3:**
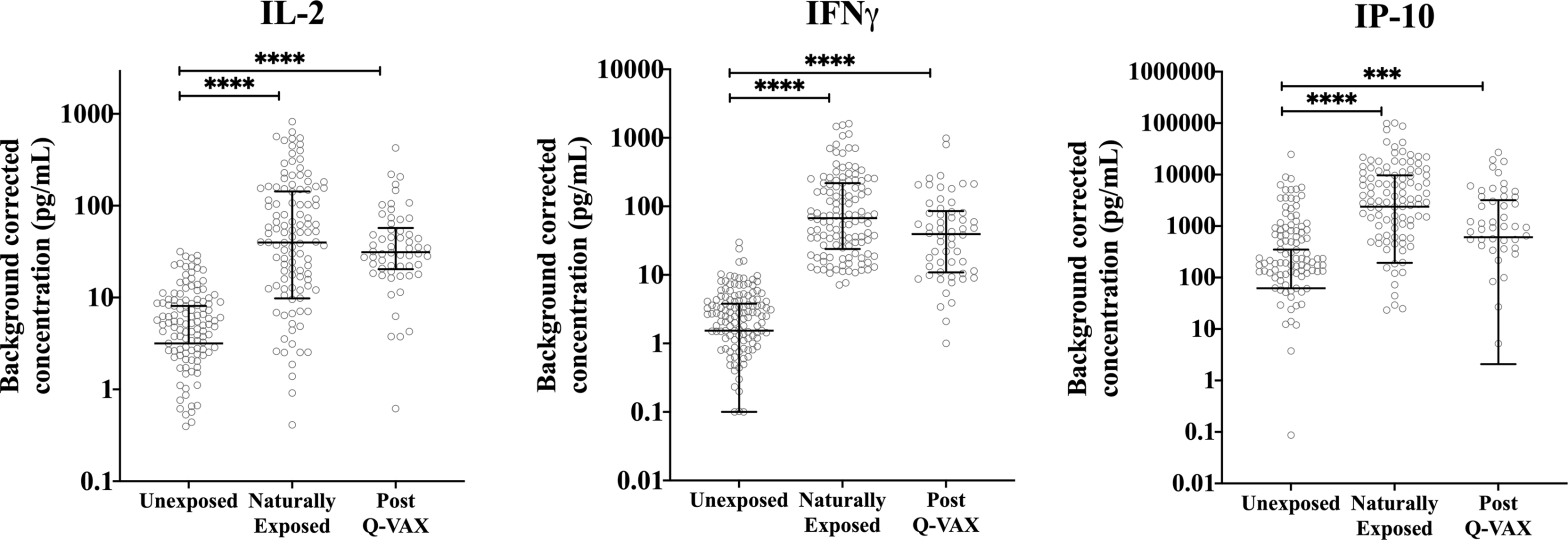
Relation of cellular responses to exposure status. Background corrected IP-10, IL-2 and IFNγ concentrations in whole blood supernatants are depicted using a scatter dot plot for unexposed, naturally exposed and vaccinated groups (n=166, n=123 and n=58, respectively). Lines and error bars show the median and interquartile range. Data are displayed on a log scale and hence zero and negative values are not represented in the graph. Cytokine responses between groups were compared using Kruskal-Wallis test followed by Dunn’s *post-hoc* multiple comparison test for nonparametric data. The asterisks (****) indicate p ≤ 0.001.

Since IgG antibody titers for phase II *C. burnetii* are a widely accepted diagnostic marker for prior *C. burnetii* infection and recent studies have demonstrated the utility of the IGRA in identifying past *C. burnetii* exposure (23, 24, 33), we decided to re-group the individuals from the Sanquin blood bank cohort, the Dutch village cohort and the Australian pre-vaccination cohort into three groups based on IGRA outcome and phase II IgG IFA titer (**Table 2**). These groups are referred to as exposure status here after. Dutch blood bank donors and study participants from the Dutch village and Australian veterinary cohorts (pre-vaccination) who were scored negative by IGRA and had a negative phase II IgG IFA titer were categorized as Unexposed. Dutch and pre-vaccination Australian participants who had a positive or borderline IGRA and/or a positive IgG antibody titer for phase II *C. burnetii* were categorized as Naturally Exposed. The Australian post-vaccination samples were treated as an independent sample set and categorized as Vaccinated. The distribution of exposure status within each clinical cohort is shown in **Figure 2B**. *In vitro* IL-2, IFNγ and IP-10 responses to C. burnetii were significantly elevated in exposed compared to unexposed participants, regardless of whether exposure occurred through infection or vaccination (**Figure 3**). Notably, in naturally exposed participants from the Dutch village cohort assessed more than a decade past infection, adaptive cytokine responses were as high (IL-2) or higher (IFNγ and IP-10) than those observed 4-5 weeks after vaccination (**Figure 3**). While some innate cytokine responses (IL-8, IL-10 and MIP1α) did not differ with exposure status, IL-1beta, IL-6 and TNFalpha responses were slightly but significantly higher in naturally exposed individuals (**Supplementary Figure 5**).

**Table 2:** Categorization of individuals based on prior exposure.

Group	N	Unexposed N	Naturally Exposed N	Vaccinated N
Dutch village cohort	95^1^	21	74	N/A
Dutch blood bank cohort	98^1^	77	21	N/A
Australian pre-vaccination cohort	96^1^	68	28	N/A
Australian post-vaccination cohort	58^2^	N/A	N/A	58

^1^Categorization based on IGRA outcome and phase II IgG IFA titer. Those who were scored negative by IGRA and had a negative phase II IgG IFA titer in 2020/21 (and 2014 and 2015 in the Dutch Village cohort) were categorized as Unexposed. Naturally Exposed was defined by a positive or borderline IGRA and/or a positive phase II IgG IFA titer in 2020/21 (and 2014 and 2015 in the Dutch Village cohort)

^2^Categorization based on Q-VAX vaccination during this study. Post-vaccination samples were treated as an independent sample set from those samples assessed pre-vaccination.

N/A, not applicable.

### High Dimensional Analysis Reveals Four Cytokine Signatures Differentially Associated With Prior Exposure

**Figure 4:**
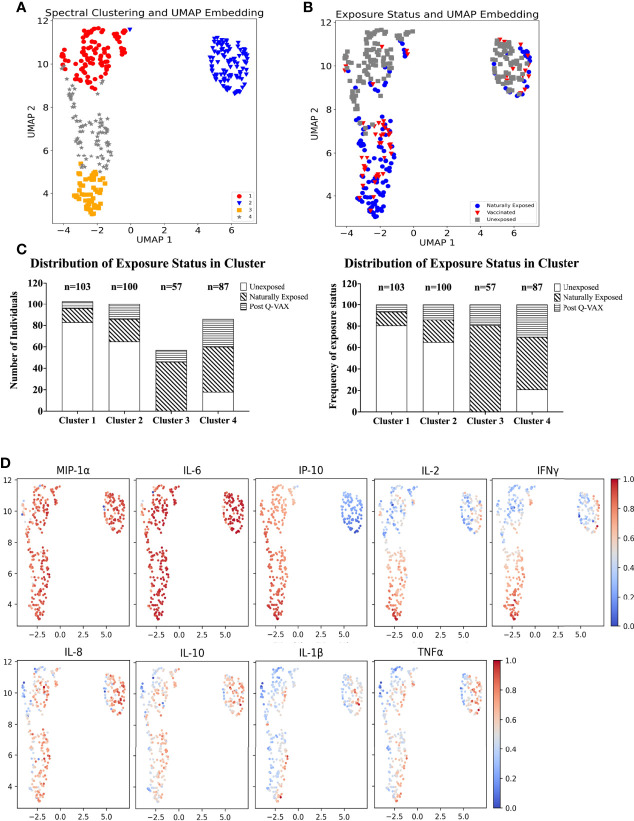
Identification of cytokine signatures by high dimensional reduction and spectral clustering of nine cytokine variables. **(A)** Scatter plot showing UMAP projections. The x and y axes represents the two UMAP coordinates after UMAP dimension reduction of all donors based on their cytokine concentrations. Each point in the plot represents an individual sample. The colour and symbol depict which cluster the sample belongs to and **(B)** the colour and symbol depict which exposure status the individual was assigned. **(C)** Stacked bar plot with absolute number of individuals per exposure status in each cluster (left) and frequency (%) of exposure status within each cluster (right). **(D)** Scatter dot plot of UMAP embeddings. Each point in the plot represents an individual sample. The colour gradient indicates from blue to red, low to high, the background corrected concentration of each cytokine. The data are min-max transformed for visualization.

UMAP was used to perform dimension reduction of all individual samples in this study based on their individual cytokine signatures. Subsequently, the embeddings were clustered using spectral clustering. Four clusters of unique cytokine signatures were identified (**Figure 4A**). Unexposed individuals were mostly confined to clusters 1 and 2, while clusters 3 and 4 were largely populated by pre-exposed individuals (by infection or vaccination). The largest proportion of all vaccinees was found in cluster 4, and cluster 3 comprised solely of individuals with prior exposure to *C. burnetii* whether through natural exposure or vaccination. (**Figures 4B, C**). Within the unexposed group of individuals (**Figure 4B**), those from the Dutch and Australian cohorts completely overlapped (data not shown). Strong MIP1α and IL-6 responses were seen in all clusters. Although IL-8, IL-10, IL-1β and TNFα had similar response magnitudes across all clusters, the individual samples with the highest response for these four cytokines were seen in cluster 2. IP-10 responses were the main driver of separation of cluster 2 (IP-10 low) from 1, 3 and 4 (IP-10 high) (**Figure 4D**), while higher IL-2 and IFNγ responses most clearly distinguished naturally exposed and vaccinated individuals (cluster 3) from unexposed ones (**Figures 4B, D**).

It is important to note that this difference in IP-10 responses arises from relatively high baseline production of IP-10 in combination with low production of IP-10 upon whole blood stimulation with *C. burnetii* by individuals in cluster 2. Individuals in cluster 1, on the other hand, had similar concentrations for IP-10 in the baseline unstimulated sample and in C. burnetii stimulated sample, while individuals in cluster 3 and 4 showed increased production of IP-10 with C. burnetii stimulation (**Supplementary Figure 6**). This pattern was seen regardless of whether individuals within each cluster were unexposed, naturally exposed or vaccinated (data not shown).

**Figure 5:**
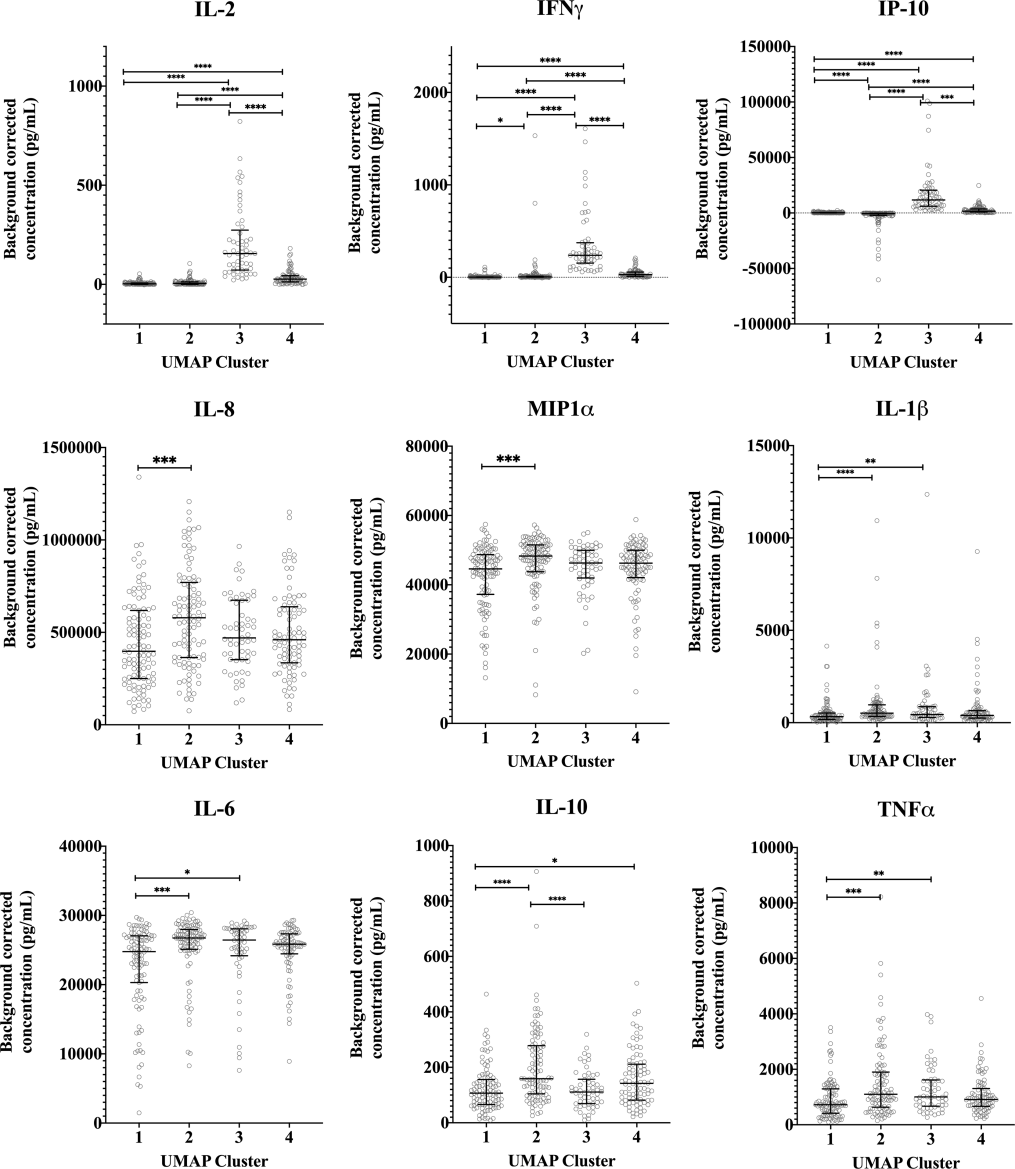
Distribution of individual cytokine responses in the four cytokine signatures. Background corrected cytokine levels in whole blood supernatants after *C. burnetii* stimulation are shown for each individual samples in each cluster for unexposed, naturally exposed and vaccinated groups (n=103, n=100, n=57 and n=87 in clusters 1, 2, 3 and 4, respectively). Lines and error bars show the median and interquartile range. Cytokine responses were compared using Kruskal-Wallis test followed by Dunn’s *post-hoc* multiple comparison test for nonparametric data. The asterisks designate the following: 0.01<p≤0.05 (*), 0.001<p≤0.01 (**), 0.0001<p≤0.001 (***) and p≤0.0001 (****).

**Figure 6:**
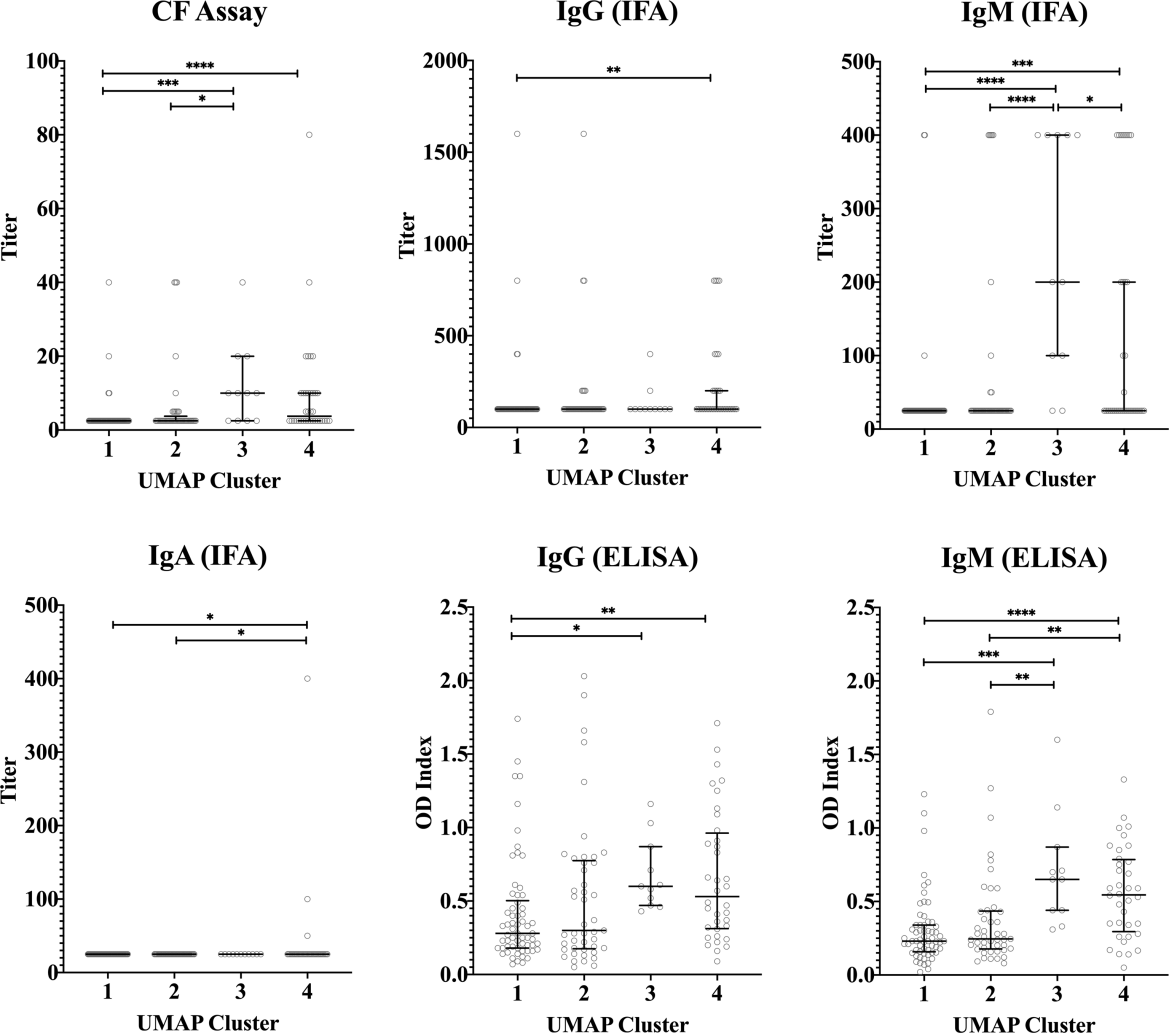
Serological response across vaccinated study participants with different cytokine signatures. Phase II IgG, IgM, IgA IFA titers, CF titers and IgG and IgM ELISA OD index in serum in each cluster (n=103, n=100, n=57 and n=87 in clusters 1, 2, 3 and 4, respectively). Data are represented using a scatter dot plot. Lines and error bars show the median and interquartile range. Serological responses were compared using Kruskal-Wallis test followed by Dunn’s *post-hoc* multiple comparison test for nonparametric data. The asterisks designate the following: 0.01<p≤0.05 (*), 0.001<p≤0.01 (**), 0.0001<p≤0.001 (***) and p≤0.0001 (****).

Median concentrations of IFNγ, IL-2 and IP-10 were highest in cluster 3 (**Figure 5**). Serological responses were compared between clusters for vaccinated Australian subjects only. While statistically significant differences were not seen in phase II IgG IFA titers across the clusters, mean phase II IgG levels by ELISA were higher in cluster 3 and 4. In addition, phase II antibody levels (IgG and IgM) determined by IFA, ELISA and CF were highest in cluster 3 (**Figure 6**).

### Individual Cytokine Responses and Cytokine Signature Patterns Do Not Associate With Clinical Symptoms During Skin Test, Vaccination or Infection

**Figure 7:**
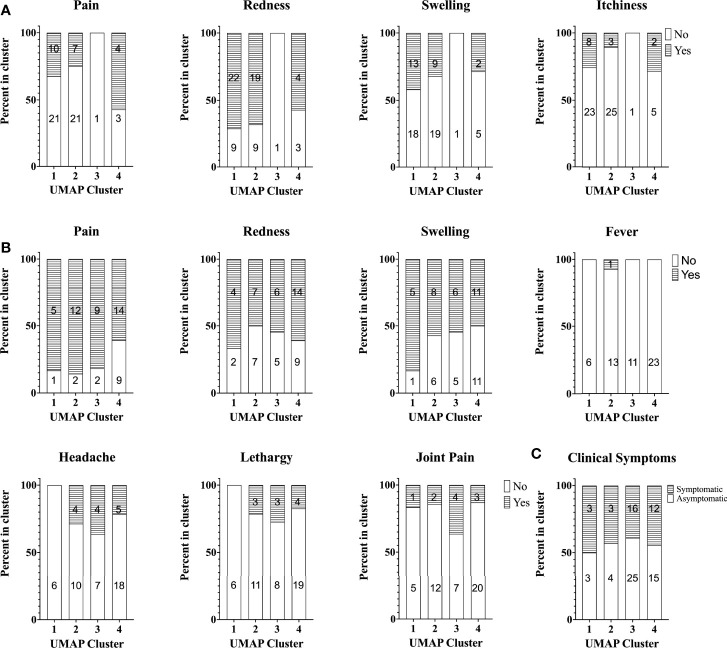
Self-reported symptoms across study participants with different cytokine signatures. Stacked bar plot of self-reported symptoms in the Australian student cohort **(A)** post-skin test (n=67) and **(B)** post-vaccination (n=54). The percentage of individuals reporting symptoms (or not) is shown per cytokine cluster. **(C)** Stacked bar plot of the percentage of individuals with self-reported symptoms during infection (asymptomatic, n=47 or symptomatic, n=34) in the Dutch Village Cohort. Numbers in columns in all graphs indicate the absolute number of individuals per cluster reporting symptoms or not.

Finally, we investigated whether there was any obvious relation between cellular responses to *C. burnetii* and clinical symptoms experienced upon Q-VAX^®^ skin test and vaccination or during natural infection. To this end, pre-vaccination cellular responses were compared prospectively to self-reported side effects upon skin test and vaccination in the Australian veterinary student cohort, while cellular responses in the Dutch village cohort were retrospectively related to previously self-reported symptoms experienced during infection. In the Australian student cohort, a digital online questionnaire was filled out by 67/96 and 54/96 students post-skin test and post-vaccination, respectively. Post-skin test, 31% reported redness, 67% pain, 36% swelling and 19% itchiness at the site of injection. Of note, none of these reactions were observed at the injection site on the 7^th^ day post-skin test when the attending physician scored the skin test. All of the participants were deemed skin test negative due to the lack of induration in the skin following palpation and thus received Q-VAX^®^ vaccination. Following Q-VAX^®^ vaccination, vaccinees commonly reported local adverse effects such as pain (74%), redness (57%) and swelling (56%). Systemic responses reported were less common and included fever (2%), headache (24%), lethargy (19%) and joint pain (19%). Naturally exposed individuals in the Dutch village cohort were categorized as symptomatic or asymptomatic based on self-reported symptoms, recorded either in 2014 (26) or earlier if individuals were officially registered (notified) through their treating physician in the Dutch national surveillance system. We found no association between skin test (**Figure 7A**) or vaccination related side effects (**Figure 7B**) and cytokine response signatures in the Australian veterinary student cohort with a comparable proportion of individuals reporting symptoms or not within each cluster. Similarly, a comparable proportion of individuals of the Dutch village cohort within each cytokine cluster had or had not experienced and re-collected symptoms during natural infection (**Figure 7C**). There were also no definitive associations between these self-reported symptoms and individual cytokine responses, including IFNγ (**Supplementary Figure 7**, data not shown for other cytokines).

## Discussion

In this study we assessed innate and adaptive cellular responses and cytokine signatures as well as humoral responses to Q-VAX^®^ vaccination and past infection with *C. burnetii* from two cohorts across the Netherlands and a cohort of Australian veterinary students. We also assessed these cytokine response signatures for any association with self-reported clinical symptoms after natural exposure or side effects after skin test and vaccination. The Dutch village cohort included in this study contracted Q fever during the outbreak between 2007-2010 in the Netherlands.

In our study, adaptive cytokine responses, as indicated by IFNγ, IL-2 and the IFNγ−inducible chemokine IP-10, were elevated in individuals with any prior exposure to *C. burnetii*, whether through vaccination or infection. Adaptive cell-mediated immune responses are known to play a crucial role in the immune system’s ability to fight *C. burnetii*, as is typical for intracellular pathogens (20, 21). Multiple studies in murine models of Q fever have shown that IFNγ knock-out or T-cell deficient animals control *C. burnetii* infection very poorly (20, 21), highlighting the essential role of these specific cellular responses. Levels of IP-10, a T-cell chemoattractant produced by monocytes and macrophages down-stream of IFNγ signalling, have been shown to be elevated in acute *C. burnetii* infected mice even before IgG antibodies against *C. burnetii* were detectable (34). It is therefore likely that these adaptive cellular responses are responsible for the strong protective nature of *C. burnetii*-specific immunity against infection after vaccination or against re-infection after natural infection (20, 35, 36).

In contrast to these three cytokines related to adaptive immunity (IFNγ, IL-2 and IP-10), no difference was seen between vaccinated and unexposed individuals for innate cytokines. Generally, all individuals showed strong innate cytokine production, consistent with previous reports and likely due to the recognition of *C. burnetii* LPS *via* TLR4, and of other ligands through TLR2 and NOD2 (37–41). Notably IL-1β, IL-6 and TNFα responses in individuals naturally exposed to viable *C. burnetii* were slightly but significantly higher when compared to unexposed individuals or to those inoculated with Q-VAX^®^ containing only formalin inactivated *C. burnetii*. The biological significance of this small difference detected in an *in vitro* re-stimulation assay is unclear. It does however raise the question of whether *in vivo* exposure to viable (but not inactivated) *C. burnetii* leads to any (pro-longed) ‘training’ of myeloid cells such as has been described for other pathogens (42, 43), a point which requires further investigation.

One potential limitation of the present study of *C. burnetii*-specific cytokine profiles is that all stimulations were conducted with heat-killed *C. burnetii.* In this context, it has previously been reported in chronic Q fever patients that *in vitro* stimulation of PBMCs from these individuals with live *C. burnetii* induces lower innate, but comparable adaptive cytokine responses, relative to stimulation by heat-killed *C. burnetii* (41). This is possibly due to the immunomodulating effects of *C. burnetii* itself, which can interfere with TLR2 and TLR4 activation (44, 45) and reduce the expression of genes related to innate immunity (46). It is not yet known whether such a difference in innate cytokine production in response to viable versus inactivated *C. burnetii* is observed in all individuals equally, regardless of prior exposure status.

Based on the Q-detect™ IGRA (24), 74% of the Australian veterinary students had IFNγ responses four weeks after vaccination. This is generally comparable to earlier studies of the Australian Q-VAX^®^ vaccine conducted in Australia and the Netherlands, which have reported *C. burnetii*-specific IFNγ responses in >75% of vaccinees within two weeks of vaccination (47) and in 63% of vaccinees (48) six months post-vaccination. Of note, the proportions are not directly comparable, both due to different cut-offs for positivity (2 pg/mL (47), 32 pg/mL (48) and 10 pg/mL in the current study) as well as technical differences between the studies during both stimulation and cytokine detection. In participants with prior exposure to *C. burnetii* by either infection or vaccination, *C. burnetii*-specific IL-2 production was clearly increased and there was a strong positive correlation between IL-2 and IFNγ production, which is in line with a prior report looking at naturally exposed individuals (49). In fact, of all the cytokines measured in our study, IL-2 responses provided the clearest delineation between pre-vaccinated and post-vaccinated populations.

Also consistent with prior studies of Q-VAX^®^, phase II IgG, IgM (by IFA and ELISA) and CF titers were significantly elevated post-vaccination (23, 50–52). In our study, four weeks post-vaccination the majority of vaccinees were positive for phase II IgG (82%), IgM (72%), and CF antibodies (52%). Consistent with known lags in the emergence of phase I antibodies (7, 8, 14) phase I IgM by IFA was detectable only in 2/58 vaccinees (3%) at four weeks post-vaccination with Q-VAX^®^.

Interestingly, we noted that there was a blunted increase in cellular responses following Q-VAX^®^ vaccination in participants who had prior exposure based on pre-existing IGRA responses, and especially in those with the highest pre-vaccination IGRA responses. This could reflect redistribution of circulating *C. burnetii*-specific memory T-cells to the tissue/vaccination site following Q-VAX^®^ vaccination, leading to apparently reduced responses in circulation observed at 4-5 weeks after vaccination in these individuals. Alternatively, IFNγ producing T-cells might not be responsive to further expansion or cytokine production. Not surprisingly, these same individuals also had a similarly blunted IP-10 response. In contrast, IL-2 responses after vaccination increased similarly regardless of whether participants were IGRA positive or negative pre-vaccination. This raises the questions whether different populations of T-cells might be responsible for *C. burnetii*-specific IFNγ and IL-2 responses, or whether IL-2 production in circulating effector memory cells might be less pronounced and hence more responsive to re-induction upon re-exposure.

High dimensional analysis of the cytokine data identified four clusters of individuals based on different signatures of cytokine responses. Clustering was largely driven by adaptive cytokine responses, namely IFNγ, IL-2 and IP-10. Distribution of individuals among these clusters was at least partially associated with prior exposure status of individuals as determined based on IGRA and/or serology assessments. Previously infected and vaccinated individuals were predominantly found in clusters 3 and 4 and unexposed individuals in clusters 1 and 2. In particular, we identified a group of individuals with high *C. burnetii*-induced levels of IFNγ, IL-2 and IP-10 (cluster 3), all of whom were either naturally exposed or vaccinated. This also raises the question whether individuals in cluster 3 would have a stronger degree of protection from *C. burnetii* infection or Q fever disease in future encounters. Adaptive responses have been shown to be required in controlling *C. burnetii* infection (19–22). However, the correlation between the intensity of these responses and (re)-infection after vaccination (or natural infection) has not yet been studied, in part because infection following Q-VAX^®^ vaccination is uncommon (36, 51, 53). Taken together, these cytokine response signature data suggest clear correlation between prior exposure and the combined strong response of all three adaptive cytokines. However, both naturally exposed and vaccinated individuals could also be found in all three other clusters, showing that prior exposure cannot be uniquely assigned to a single cytokine response signature.

Individuals in cluster 2 exhibited higher baseline levels of IP-10 production (i.e in negative control stimulations) than were observed for individuals in other clusters. These individuals further exhibited no or very little increase in IP-10 production in response to *C. burnetii* stimulation, which was a defining parameter of cluster 2. Although individuals in cluster 2 were largely from the unexposed group, 16% of naturally exposed and 24% of vaccinated volunteers were also seen in this cluster. IFNγ and IL-2 adaptive responses were seen amongst cluster 2 individuals with prior exposure to *C. burnetii* whether through infection or Q-VAX^®^ vaccination, although to a lesser extent than observed in cluster 3. At the same time, these samples showed high innate cytokine responses, and specifically higher than in cluster 1. This pattern of high baseline IP-10, low *C. burnetii*-induced IP-10 and high *C. burnetii*-induced innate cytokine production suggests that although the cluster 2 samples from individuals with prior exposure have a myeloid/macrophage driven response to *C. burnetii*, their adaptive responses resulting from prior exposure to *C. burnetii* are not leading to downstream enhancement of IP-10 secretion. One possible hypothesis for the strong innate response to *C. burnetii* in cluster 2 individuals is potential prior exposure to other pathogens inducing an epigenetic or metabolic re-programming resulting in a general ‘training’/enhanced responsiveness of innate immune cells (43). However, the question remains why IFNγ failed to enhance downstream IP-10 secretion in the cluster 2 indviduals with adaptive responses to *C. burnetii*, and what implications this might have for their protection from *C. burnetii* (re-)infection.

Notably, the Australian cohort included some participants who were IGRA positive pre-vaccination and thus considered pre-exposed. However, since all volunteers in the Australian cohort were judged negative by skin test and serology, and hence were vaccinated, no comparison was possible between cytokine responses in participants with a positive or negative skin test as a different measure of cellular immunity, as has been conducted elsewhere (17). Nevertheless, we were able to assess innate and adaptive cytokine responses and signatures prospectively or retrospectively in relation to self-reported clinical symptoms during infection or upon skin test or vaccination. Neither showed any association, with comparable proportions of individuals reporting symptoms within each cytokine signature. For *C. burnetii*-specific IFNγ, this is in contrast to a prior study in Dutch Q-VAX vaccinees, in which pre-vaccination *C. burnetii*-specific IFNγ responses correlated with local adverse events (17). However, these Dutch vaccinees were considerably older than the Australian student cohort in this present study. In addition, the study of Dutch Q-VAX vaccinees was conducted during an ongoing outbreak in The Netherlands and hence the volunteers were likely more recently exposed to *C. burnetii*, with potentially greater proportions of effector rather than central memory T-cells capable of immediately contributing to the skin test and vaccination response.

*C. burnetii*-specific IFNγ and IP-10 responses in naturally exposed study participants more than a decade past infection were as high or higher than those observed 4-5 weeks after vaccination. This could be due, at least partially, to a selection bias in the longitudinally followed Dutch village cohort. This cohort originated from a larger group initially selected for a different study in 2015 (27), in which preference was given to participants with strong responses to whole heat-killed *C. burnetii* by IGRA to maximize the potential to also detect *C. burnetii* epitope-specific T-cells. Nevertheless, the present data show that these participants continue to have remarkably high levels of cellular as well as serological responses 10-14 years after infection, highlighting the durability of *C. burnetii*-induced adaptive immunity. To the best of our knowledge, such broad and long-term cytokine responses, 10-14 years after exposure, have not previously been studied in individuals with a history of Q fever.

In conclusion, we show here that *C. burnetii* unexposed individuals and those exposed by natural exposure or vaccination fall into four clusters with distinct cytokine response signatures. Prior exposure status was partially associated with these signatures, but could not be clearly assigned to a single cytokine response signature. The adaptive cytokines IFNγ and IL-2 as well as the IFNγ-induced chemokine IP-10 were the key correlates of prior exposure by infection and vaccination. In addition, a subset of innate cytokine responses were increased following natural exposure but not vaccination with inactivated *C. burnetii*. One subgroup of individuals - whether exposed or unexposed - was defined by the presence of strong innate responses and absent IP-10 induction in response to *C. burnetii*, paired with high baseline IP-10-level. Finally, neither innate nor adaptive cytokine responses were associated retrospectively with clinical symptoms during infection or prospectively with side effects post-vaccination.

## Data Availability Statement

The original contributions presented in the study are included in the article/**Supplementary Material**. Further inquiries can be directed to the corresponding authors.

## Ethics Statement

The studies involving human participants were reviewed and approved by University of Queensland Human Research Ethics Committee (Brisbane, Australia) and Medical Ethical Committee Brabant (Tilburg, Netherlands). The patients/participants provided their written informed consent to participate in this study.

## Author Contributions

AG, AES, AS, JR, MP, RC and SG conceptualized and designed the study and experiments. AS and RC coordinated enrollment and sample collection of the Dutch and Australian cohorts, respectively. SG had clinical oversight of the Australian cohort, performed and interpreted the skin test and vaccination. Experiments were performed by AS, SW, JE and PH. Survey Monkey data were collected and compiled by RC. Data were analyzed and integrated by AS, GM, RD, SRP. AG, AES, JR, MP, RC and SG acquired funding and supervised research activities. AS and SRP interpreted data and wrote the manuscript. All authors critically reviewed and approved the final version of the manuscript.

## Funding

This work was supported by grant HDTRA1201006 from the US Defense Threat Reduction Agency (www.dtra.mil), awarded to Massachusetts General Hospital, with MCP as Lead Principal Investigator.

## Conflict of Interest

AG is CEO and AS was employed at Innatoss Laboratories, and SW, PH, JE and JR are employees of Sullivan Nicolaides Pathology, both providing diagnostic screening for Q fever.

The remaining authors declare that the research was conducted in the absence of any commercial or financial relationships that could be construed as a potential conflict of interest.

## Publisher’s Note

All claims expressed in this article are solely those of the authors and do not necessarily represent those of their affiliated organizations, or those of the publisher, the editors and the reviewers. Any product that may be evaluated in this article, or claim that may be made by its manufacturer, is not guaranteed or endorsed by the publisher.
