# Supplementary material for: Natural Exposure- and Vaccination-Induced Profiles of Ex Vivo Whole Blood Cytokine Responses to Coxiella burnetii
Source: Front Immunol. 2022 Jun 23;13:886698. doi: 10.3389/fimmu.2022.886698 (PMC9259895; doi:10.3389/fimmu.2022.886698)

Supplementary Material

Supplementary Figures

Supplementary Figure 1. Positive and negative control data and *C. burnetii*-specific IFNγ responses for all four cohorts. (A) Background concentrations of IFNγ in whole blood plasma supernatants after negative control stimulation. **(B)** IFNγ concentrations in whole blood plasma supernatants after stimulation with SEB. **(C)** Background corrected *C. burnetii*-specific IFNγ concentration in whole blood plasma supernatants. Data for the Dutch village cohort (n=95), Dutch blood bank donors (n=98), Australian students pre (n=96) and post Q-VAX® vaccination (n=58) are shown. Data are displayed on a log scale and hence zero and negative values are not represented in the graph. Individual data are represented using a scatter dot plot. Lines and error bars show the median and interquartile range. Cytokine responses between groups were compared using Kruskal-Wallis test followed by Dunn's post-hoc multiple comparison test for nonparametric data. The asterisks designate the following: 0.01<p≤0.05 (*), 0.001<p≤0.01 (**) and p≤0.0001 (****).

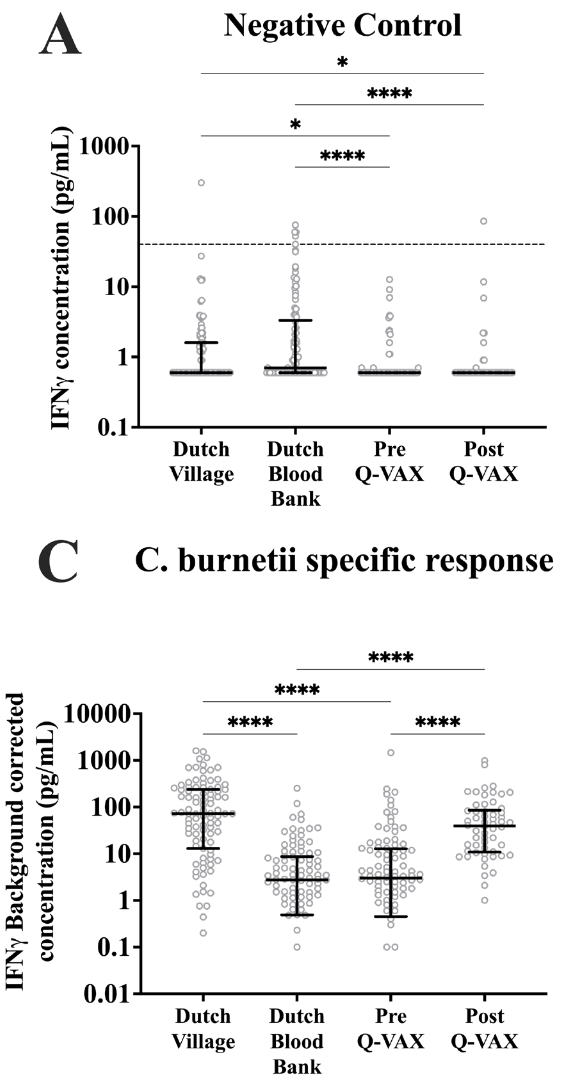

Supplementary Figure 2. Vaccination-induced changes in innate cytokine responses to *C. burnetii*. Background corrected innate cytokine levels in whole blood supernatants after *C. burnetii* stimulation conducted prior to vaccination and at 4-5 weeks post-vaccination. Data are shown only for those participants that attended the post-vaccination follow-up visit (n=58 vaccinees). Data are displayed on a log scale and hence zero and negative values are not represented in the graph. Individual data are represented using a scatter dot plot. Lines and error bars show the median and interquartile range. Pre- and post-vaccination responses were compared using Wilcoxon matched-pairs signed rank test. No comparisons were significant (all p>0.05).

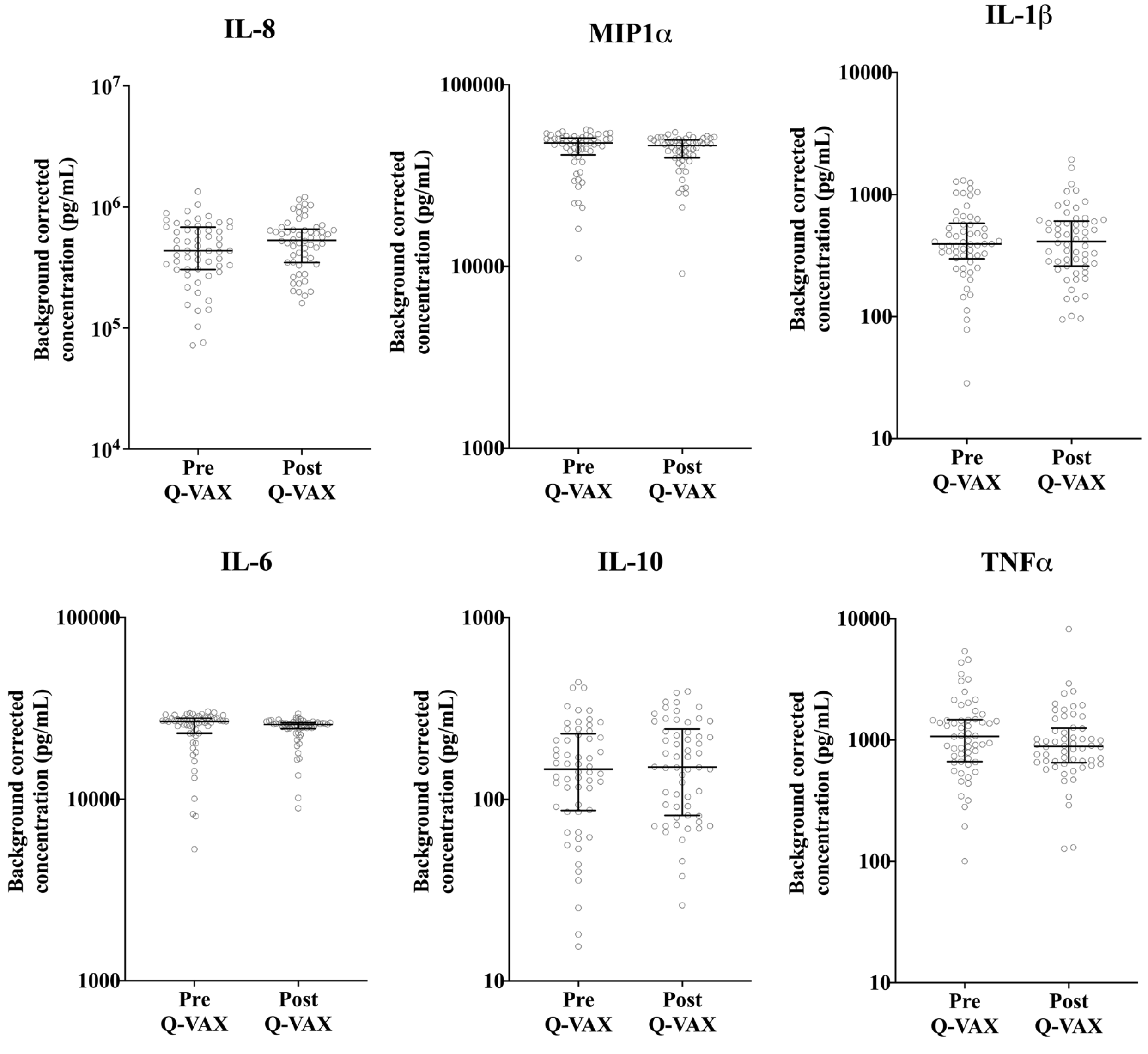

Supplementary Figure 3. Influence of pre-existing immunity on Q-VAX®-induced *C. burnetii*-specific cytokine responses. Matched pre-vaccination and post-vaccination adaptive cytokine levels (IFNγ, IP-10, IL-2) in whole blood plasma supernatants after *C. burnetii* stimulation are shown for samples from all vaccinated participants (n=58; left column) or samples from participants that were at pre-vaccination either IGRA positive (n=21, middle column) or IGRA negative (n=37, right column). Lines connect pre- and post-vaccination samples per individual. Pre- and post-vaccination responses were compared using Wilcoxon matched-pairs signed rank test. The asterisks designate the following: 0.05<p≤0.01 (**), 0.0001<p≤0.001 (***) and p≤0.0001 (****).

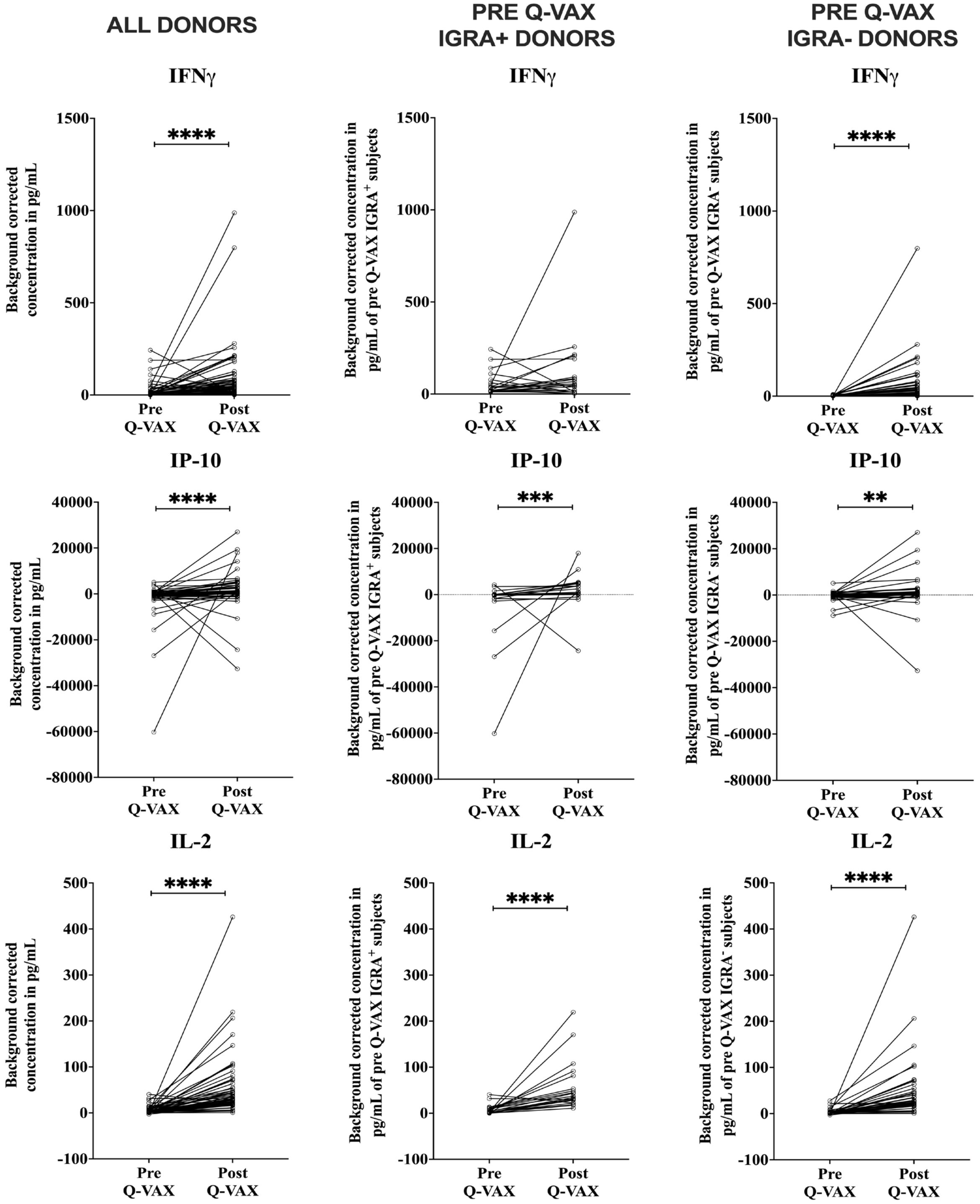

Supplementary Figure 4. Correlation of cellular and serological responses in subjects after Q-VAX® vaccination. Correlation matrix with blue to red color gradient indicating -1 to +1 values, which indicate the Spearman Rho. Spearman Rho values inside the square are entered only for p < 0.05.

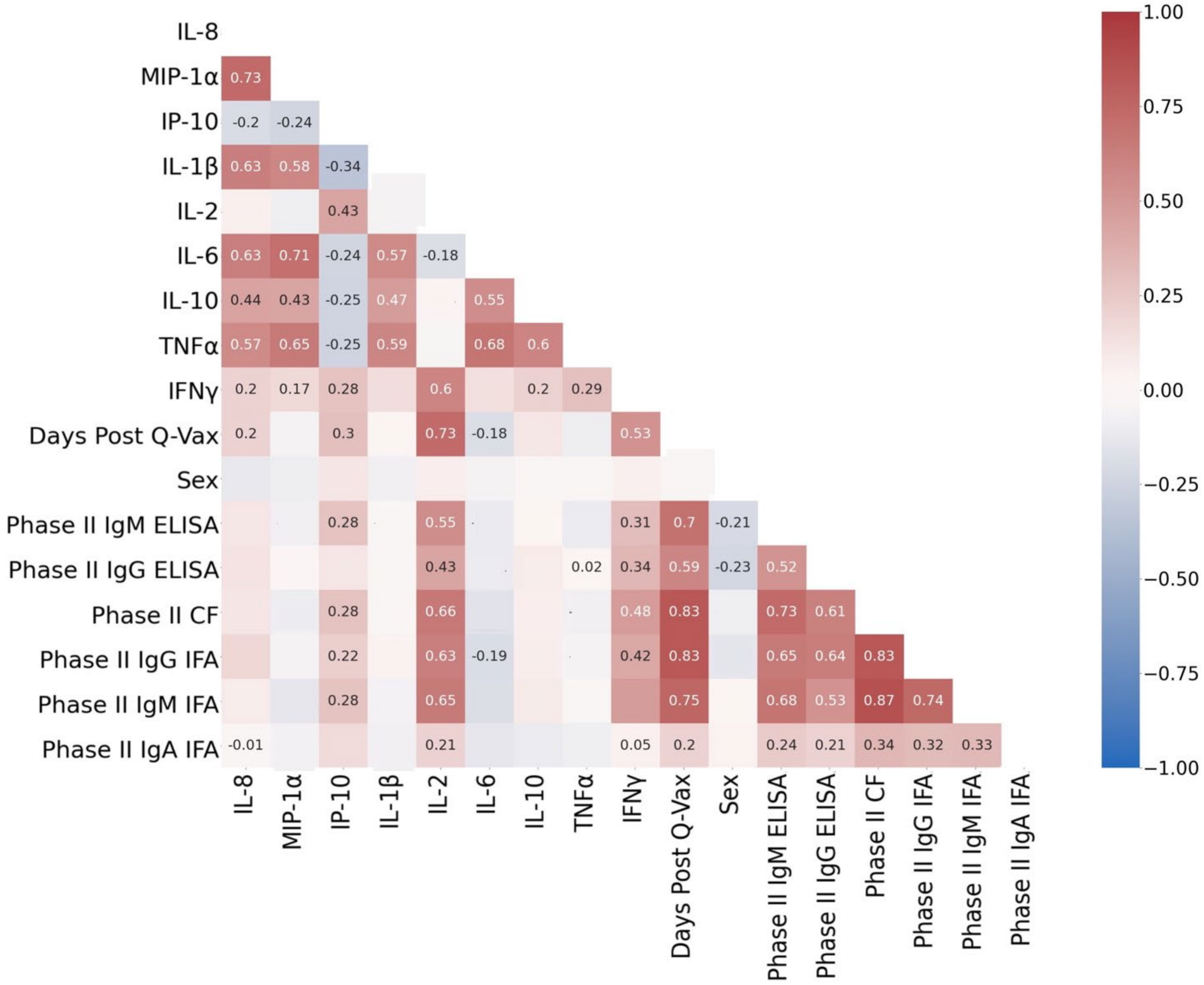

Supplementary Figure 5. Association of cytokine responses with prior *C. burnetii* exposure status. Background corrected innate cytokine levels in whole blood supernatants after *C. burnetii* stimulation are depicted using a scatter dot plot for unexposed, naturally exposed, and vaccinated groups (n=166, n=123 and n=58, respectively). Lines and error bars show the median and interquartile range. Data are displayed on a log scale and hence zero and negative values are not represented in the graph. Groups were compared using Kruskal-Wallis test followed by Dunn's post-hoc multiple comparison test for nonparametric data. The asterisks designated the following: 0.01<p≤0.05 (*) and 0.001<p≤0.01 (**).

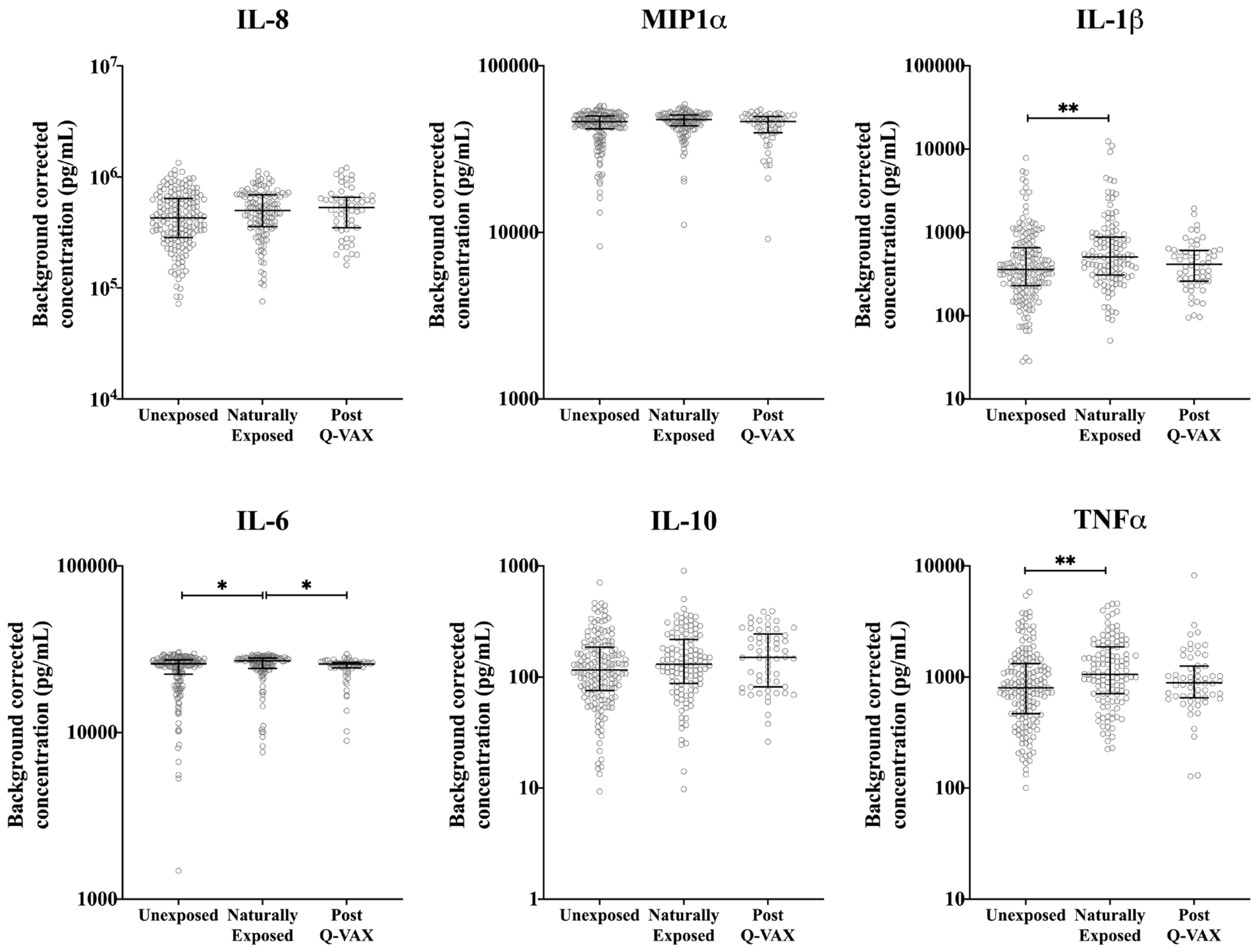

Supplementary Figure 6. Basal and *C. burnetii*-induced IP-10 release for individuals in the four cytokine signature clusters. IP-10 concentrations in whole blood supernatants from whole blood negative control and *C. burnetii* stimulations are shown for individuals in clusters 1, 2, 3 and 4 (n=103, n=100, n=57 and n=87, respectively). Lines connect negative control and *C. burnetii* stimulated samples per individual. Responses were compared using Wilcoxon matched-pairs signed rank test. The asterisks designate the following: p≤0.0001 (****).

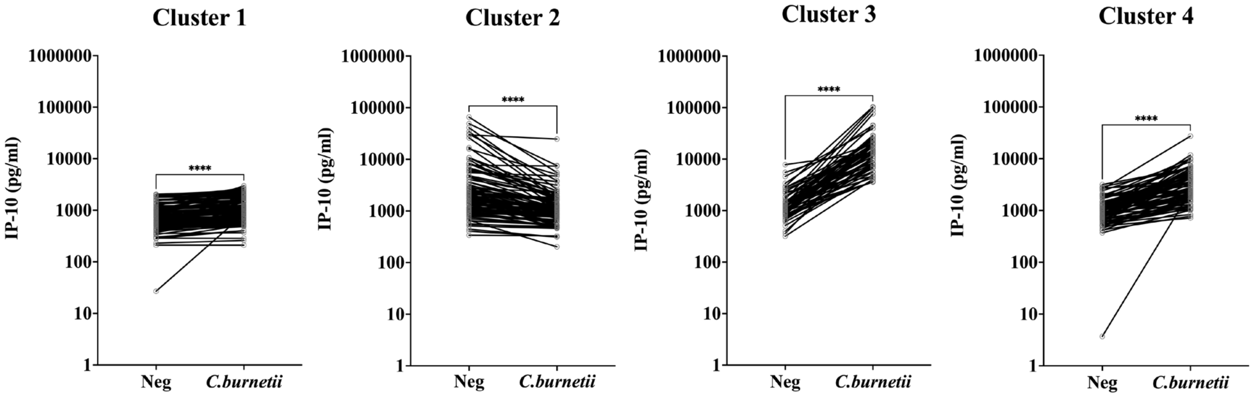

Supplementary Figure 7. Baseline *C. burnetii* specific IFNγ responses in individuals with and without self-reported symptoms in response to Q-VAX® skin test and vaccination. Background corrected *C. burnetii*-specific IFNγ responses at baseline are shown for individuals without (No) and with (Yes) self-reported symptoms in the Australian student cohort **(A)** post-skin test (n=67) and **(B)** post-vaccination (n=54) and for **(C)** self-reported symptoms (Yes, n=34) or no symptoms (No, n=47) during past infection in the Dutch Village Cohort. Data are displayed on a log scale and hence zero and negative values are not represented in the graph. Individual data are represented using a scatter dot plot. Lines and error bars show the median and interquartile range. Responses were compared using Mann-Whitney test for non-parametric data. No comparisons were significant (all p>0.05).

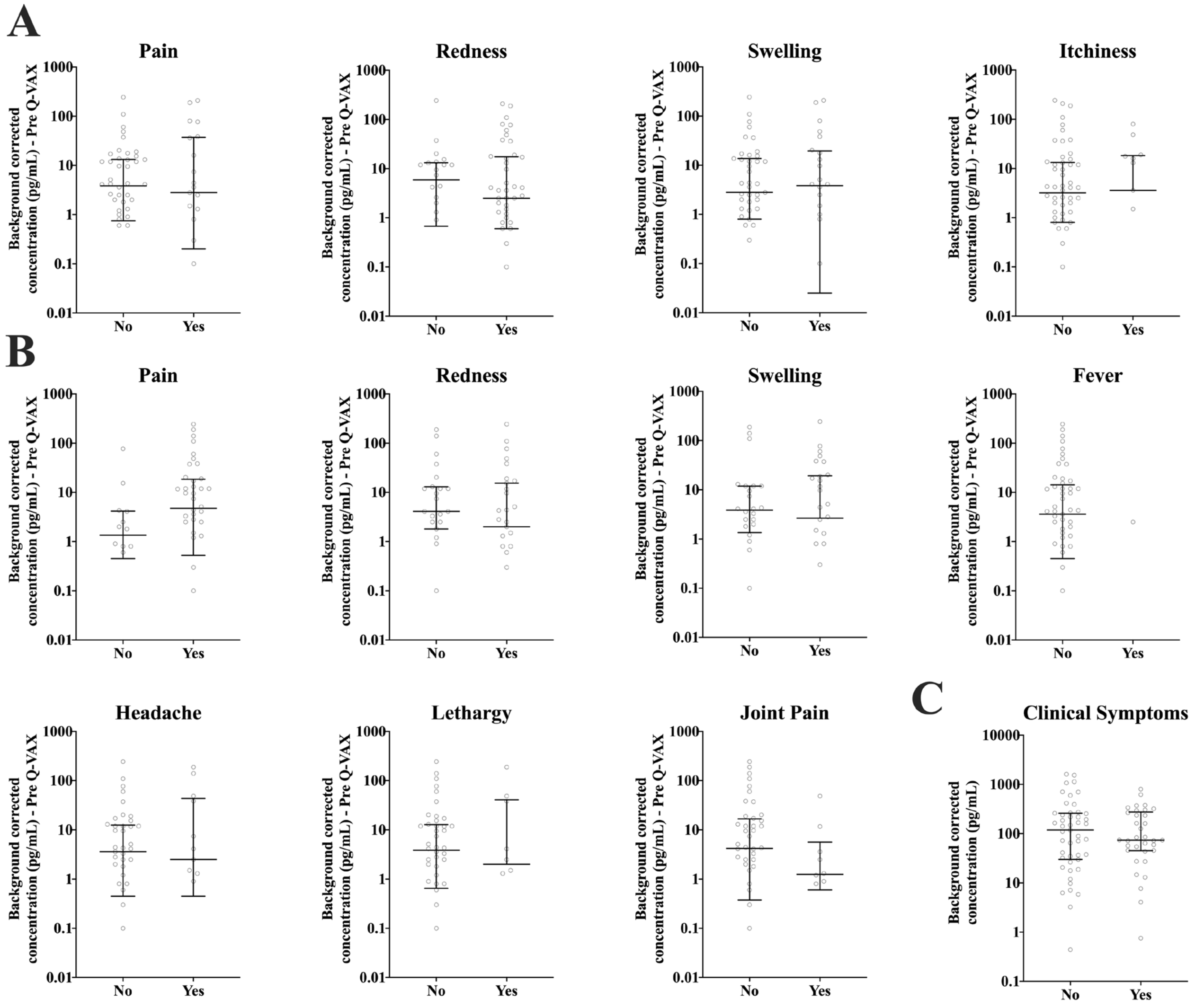

Supplement: Supplementary file 1 [file DataSheet_1.pdf]
